# Diffuse ^18^F-fluorodeoxyglucose accumulation in the bone marrow of a patient with haemophagocytic lymphohistiocytosis due to Hodgkin lymphoma

**DOI:** 10.1136/bcr-2016-217555

**Published:** 2016-10-28

**Authors:** Shu Harada, Tsutomu Shinohara, Keishi Naruse, Hisanori Machida

**Affiliations:** 1 Division of Pulmonary Medicine, National Hospital Organization Kochi Hospital, Kochi, Japan; 2 Department of Clinical Investigation, National Hospital Organization Kochi Hospital, Kochi, Japan; 3 Division of Pathology, National Hospital Organization Kochi Hospital, Kochi, Japan

## Description

**Figure 1:**
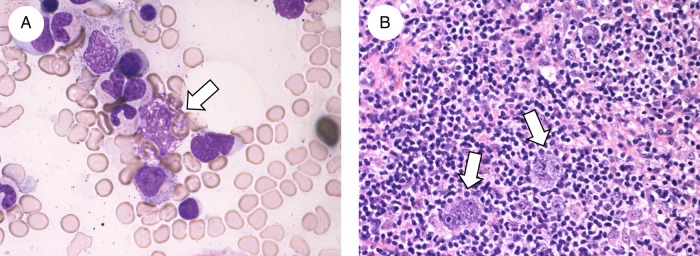
Pathological findings. (A) Bone marrow aspirate smear showing macrophages laden with erythrocytes (arrow). (B) Abdominal lymph node biopsy showing scattered large atypical multinucleated cells (Reed-Sternberg cells; arrows) in prominent small lymphocytes.

**Figure 2:**
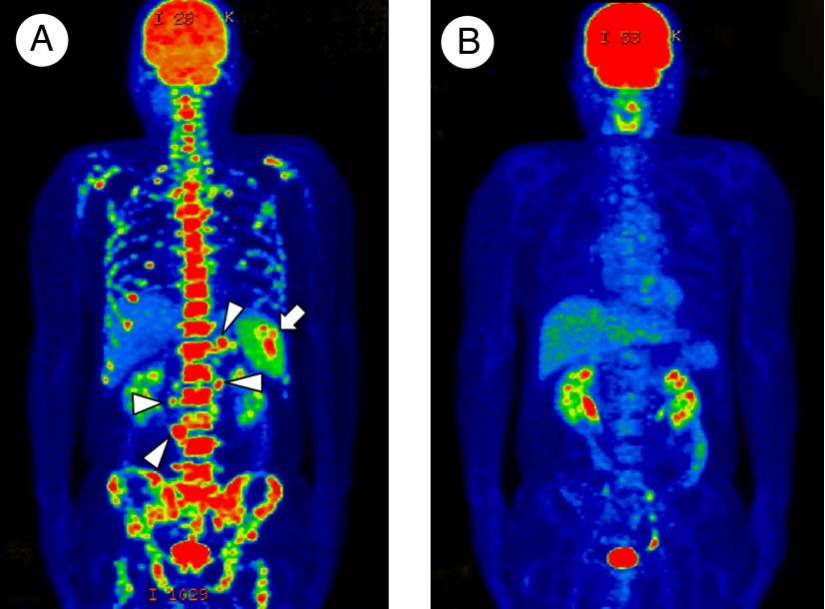
^18^F-fluorodeoxyglucose positron emission tomography before (A) and after (B) the four courses of ABVD (doxorubicin, bleomycin, vinblastine and dacarbazine) chemotherapy. (A) Splenic (arrow) and multiple abdominal lymph node (arrowheads) lesions and diffuse accumulation within bones such as vertebras and pelvis. (B) Disappearance of all abnormal accumulations.

A 53-year-old man was referred to our hospital due to malaise with intermittent high fever. Thoracoabdominal CT scan revealed abdominal lymphadenopathy with splenomegaly. Hepatic disorder (aspartate transaminase 47 IU/L; alanine transaminase 53 IU/L), peripheral blood cytopaenia (red blood cell count 370×10^4^/μL; haemoglobin 11.0 g/dL; haematocrit 32.9%; platelet count 7.9×10^4^/μL), hypertriglyceridaemia (184 mg/dL), hyperferritinaemia (4111 ng/mL) and an increased serum soluble interleukin-2 receptor level (4450 U/mL) were observed. Bone marrow (BM) aspiration showed erythrocyte phagocytosis by macrophages (figure 1A). However, no atypical cells were detected. Additional BM trephine biopsy was not performed. Pathological examination of a laparoscopic lymph node biopsy revealed Reed-Sternberg cells (figure 1B), and atypical cells were positive for CD30 but not for CD3, CD20 or CD79a indicating nodular sclerosis Hodgkin lymphoma (HL). A diagnosis of lymphoma-associated haemophagocytic lymphohistiocytosis (LA-HLH) was established. ^18^F-fluorodeoxyglucose positron emission tomography (FDG-PET) performed for the staging showed splenic and multiple abdominal lymph node lesions and diffuse accumulation within the bones such as the vertebrae and pelvis (figure 2A). After four courses of ABVD (doxorubicin, bleomycin, vinblastine and dacarbazine) chemotherapy, FDG-PET showed the disappearance of all abnormal accumulations (figure 2B). Involved-field radiation therapy for abdominal lesions was sequentially added.

Patients with HLH with FDG-PET images indicating multiple lymphadenopathy and/or patchy multiple bone lesions may appear to be LA-HLH with BM involvement (BMI).^1^ However, in comparison with BM examinations, FDG-PET can frequently show false-positive for the evaluation of BMI in patients with LA-HLH because HLH can induce diffuse hypermetabolism in BM reflecting a systemic cytokine storm.^2^ Previously, several cases of HLH due to non-HL with diffuse FDG accumulation in BM were reported.^2–3^ Since we performed BM aspiration only without BM trephine biopsy, skeletal and/or BM HL involvement cannot be fully ruled out. However, there is a possibility that HLH due to HL also can induce diffuse hypermetabolism in BM.

**Learning points**

- ^18^F-fluorodeoxyglucose positron emission tomography (FDG-PET) has become a standard tool for the initial staging and reassessment of Hodgkin lymphoma, which is generally characterised by contiguous lymph node involvement.
- Haemophagocytic lymphohistiocytosis, which is the uncontrolled activation of lymphocytes and macrophages caused by various diseases including malignant lymphoma, can induce diffuse hypermetabolism in the bone marrow reflecting a systemic cytokine storm.
- Recognition of false-positive findings on FDG-PET is necessary for the evaluation of bone marrow involvement in order to accurately determine the stage of Hodgkin lymphoma, which requires bone marrow examination.
